# Regulating Astrocytes via Short Fibers for Spinal Cord Repair

**DOI:** 10.1002/advs.202406742

**Published:** 2024-08-09

**Authors:** Qianyi Li, Shuaiyun Gao, Yang Qi, Nuo Shi, Zhenzhen Wang, Qimanguli Saiding, Liang Chen, Yawei Du, Bo Wang, Wenfei Yao, Bruno Sarmento, Jie Yu, Yiming Lu, Juan Wang, Wenguo Cui

**Affiliations:** ^1^ Department of Orthopaedics Shanghai Key Laboratory for Prevention and Treatment of Bone and Joint Diseases Shanghai Institute of Traumatology and Orthopaedics Ruijin Hospital Shanghai Jiao Tong University School of Medicine Shanghai 200025 P. R. China; ^2^ Department of Emergency Ruijin Hospital Shanghai Jiaotong University School of Medicine Shanghai 200025 P. R. China; ^3^ Pˆole Sino‐Franc¸ais de Recherches en Sciences du Vivant et G´enomique Shanghai 200025 P. R. China; ^4^ International Laboratory in Cancer Aging and Hematology Shanghai Jiao Tong University School of Medicine/Ruijin Hospital/CNRS/Inserm/Cote d'Azur University Shanghai 200025 P. R. China; ^5^ Peterson's Lab Shanghai 200030 P. R. China; ^6^ I3‐Instituto de Investigação e Inovação Em Saúde and INEB‐Instituto de Engenharia Biomédica Universidade Do Porto Rua Alfredo Allen 208 Porto 4200‐135 Portugal; ^7^ IUCS‐Instituto Universitário de Ciências da Saúde CESPU Rua Central de Gandra 1317 Gandra 4585‐116 Portugal; ^8^ Division of Critical Care Nanxiang Hospital of Jiading District Shanghai 201802 P. R. China

**Keywords:** electrospun fibers, neural repair, reactive astrogliosis, short fibers, spinal cord injury

## Abstract

Reactive astrogliosis is the main cause of secondary injury to the central nerves. Biomaterials can effectively suppress astrocyte activation, but the mechanism remains unclear. Herein, Differentially Expressed Genes (DEGs) are identified through whole transcriptome sequencing in a mouse model of spinal cord injury, revealing the VIM gene as a pivotal regulator in the reactive astrocytes. Moreover, DEGs are predominantly concentrated in the extracellular matrix (ECM). Based on these, 3D injectable electrospun short fibers are constructed to inhibit reactive astrogliosis. Histological staining and functional analysis indicated that fibers with unique 3D network spatial structures can effectively constrain the reactive astrocytes. RNA sequencing and single‐cell sequencing results reveal that short fibers downregulate the expression of the *VIM* gene in astrocytes by modulating the “ECM receptor interaction” pathway, inhibiting the transcription of downstream Vimentin protein, and thereby effectively suppressing reactive astrogliosis. Additionally, fibers block the binding of Vimentin protein with inflammation‐related proteins, downregulate the NF‐κB signaling pathway, inhibit neuron apoptosis, and consequently promote the recovery of spinal cord neural function. Through mechanism elucidation‐material design‐feedback regulation, this study provides a detailed analysis of the mechanism chain by which short fibers constrain the abnormal spatial expansion of astrocytes and promote spinal cord neural function.

## Graphical abstract

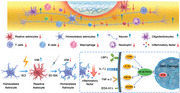

## 1 Introduction

The repair of spinal cord nerve injury has always been an important scientific problem in the field of neuroscience and regenerative medicine.^[^ ^1^, ^2^ ^]^ The complex interaction between neurons and neuroglia cells further complicates the process of nerve repair. Astrocytes, in particular, exhibit abnormal spatial expansion behavior (known as reactive astrogliosis) after nerve injury.^[^ ^3^, ^4^, ^5^ ^]^ Their persistent abnormal spatial encroachment mechanically compresses and damages neurons, while the continuous release of pro‐inflammatory and chemotactic factors leads to secondary damage in nerve tissues.^[^ ^6^ ^]^ This worsens the microenvironment for nerve regeneration, affecting the conduction and communication functions of neurons.^[^ ^7^, ^8^ ^]^ Ultimately, the disorderly growth of reactive astrocytes persistently hinders the recycling of neurotransmitters at synaptic clefts, maintenance of the blood–brain barrier (BBB), and regulation of energy homeostasis, leading to permanent neural damage.^[^ ^9^ ^]^ Therefore, inhibiting reactive astrogliosis and maintaining their spatial homeostasis is crucial for creating a microenvironment conducive to neuronal regeneration.

At present, the treatment of reducing the effects of reactive astrocytes after spinal cord injury (SCI) mainly incorporates the use of cell‐cycle inhibition drugs to eliminate reactive astrocytes.^[^ ^10^, ^11^ ^]^ However, the oral administration of such drugs often requires a high dose and frequency; moreover, nonspecific drugs are prone to induce activity on multiple targets, leading to serious side effects. Furthermore, in the aftermath of SCI, the BBB is compromised, which hinders the delivery of oral medications to the injured site and impedes effective control over reactive astrocytes.^[^ ^12^ ^]^ Therefore, in‐depth studies of molecular regulatory mechanisms post‐nerve injury are critical to uncover key gene expression, associated signaling pathways, and potential therapeutic targets in astrocytes,^[^ ^13^ ^]^ Such insights are fundamental for developing novel biomaterials capable of limiting reactive astrocytes from the initiating factors.

In recent years, various biomaterials with specific functions and characteristics have been developed to support and enhance the repair process after SCI, such as hydrogels,^[^ ^14^, ^15^ ^]^ microspheres,^[^ ^16^ ^]^ and 3D‐printed scaffolds.^[^ ^17^ ^]^ Among them, nanofibers are widely used in repairing damaged nerve tissues^[^ ^18^, ^19^, ^20^ ^]^ due to their close simulation of the natural extracellular matrix (ECM) and excellent processability.^[^ ^21^, ^22^, ^23^ ^]^ Previous studies have demonstrated that fibrous scaffolds can provide structural support, facilitate cell migration, and enhance axonal growth.^[^ ^24^, ^25^ ^]^ However, while long fibers have been traditionally used, recent studies suggest that shorter fibers might offer distinct advantages. Specifically, short fibers could be more easily integrated into the injury site, reduce inflammation, and promote a more favorable environment for neural repair.^[^ ^26^ ^]^ Despite these findings, existing research primarily focuses on the responsive behaviors of neural cells to fibers, leaving the precise mechanisms by which fibers influence neural cells still obscure.^[^ ^27^, ^28^ ^]^ Especially in the study of astrocytes, it remains to be thoroughly investigated how fibers and other biomaterials trigger regulatory mechanisms to specifically modulate their gene expression, as well as how these processes affect cellular regeneration functions and enhance neural tissue repair processes. Therefore, it is crucial to thoroughly comprehend the interaction mechanisms between biomaterials and astrocytes, as well as how these mechanisms affect the overall efficacy of neural repair for the advancement of regenerative fiber systems in clinical applications.

**Figure 1:**
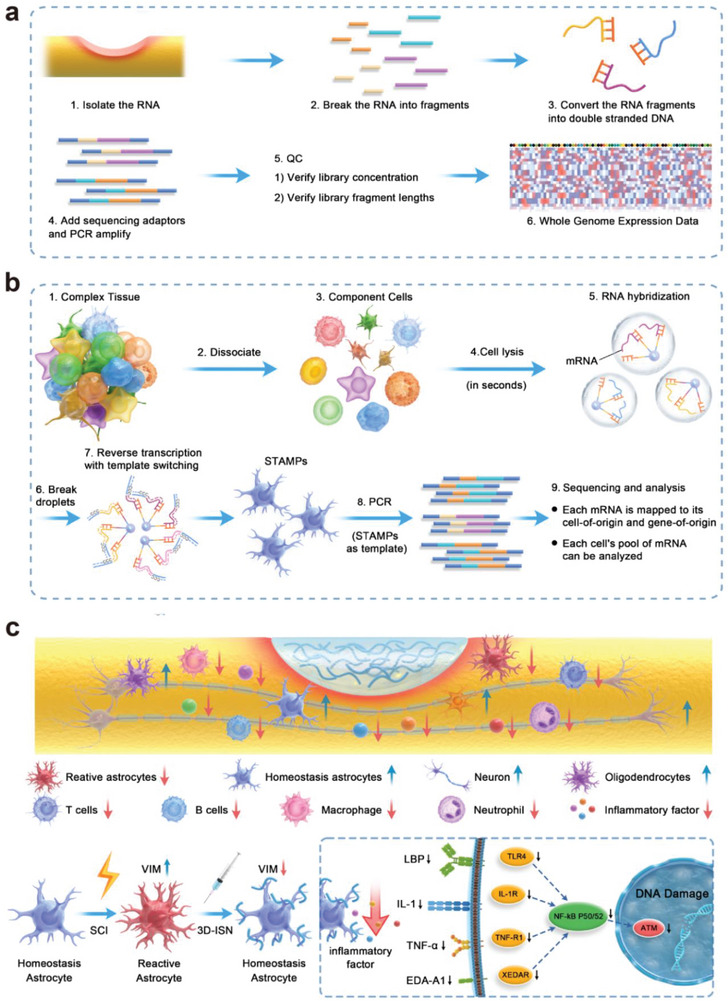
Schematic diagram of the experimental process. a) Whole transcriptome analysis of spinal cord neural tissue after injury. b) Single‐cell sequencing analysis of gene expression in astrocytes. c) Schematic diagram of the chain of mechanisms by which 3D short fibers constrain the spatial expansion of astrocytes.

In order to systematically analyze the evolution, structure, and functional regulation of astrocytes as well as their mechanistic interplay with SCI, this study established a mouse compression SCI model and analyzed the primary mechanisms and action targets of the reactive astrocytes by RNA‐Sequence technique. Meanwhile, biomaterials have been innovatively developed for the key nodes to effectively inhibit the reactive astrogliosis and the mechanism of action was illuminated (**Figure** **1**). Initially, at the transcriptional level, the protein–protein interaction (PPI) network of differentially expressed genes (DEGs) was analyzed between the SCI group and Sham group, identifying key regulatory targets. Subsequently, through an in‐depth analysis of cell components, and by combining electrospinning, high‐speed homogenization, and cross‐linking technology, injectable 3D electrospun short fibers^[^ ^29^, ^30^, ^31^ ^]^ were fabricated to be regulated by the extracellular space. In vitro, structural and functional transformations of neuroglia cells differentiating were systematically simulated and analyzed. High‐resolution imaging and molecular biology techniques quantified their morphological changes on 3D electrospun short fibers, clarifying fiber‐cell interactions. Furthermore, a mouse SCI model was used to comprehensively evaluate the effects of 3D electrospun short fibers on the growth, orientation, and intracellular signal transduction of nerve cells. Finally, through the combination of whole transcriptome and single‐cell sequencing, the study focused on the dynamic changes of astrocyte subgroups and the expression pattern of the key gene *VIM* (encoding Vimentin) during the neural repair process, comprehensively revealing the regulatory mechanism of the 3D short fibers on the signaling pathways that constrained the abnormal spatial expansion of astrocytes. Overall, this study provides an important biological basis for therapeutic strategies for neural repair.

## 2 Results and Discussion

### 2.1 Transcriptome Analysis of Spinal Cord Compression Injury in Mice

**Figure 2:**
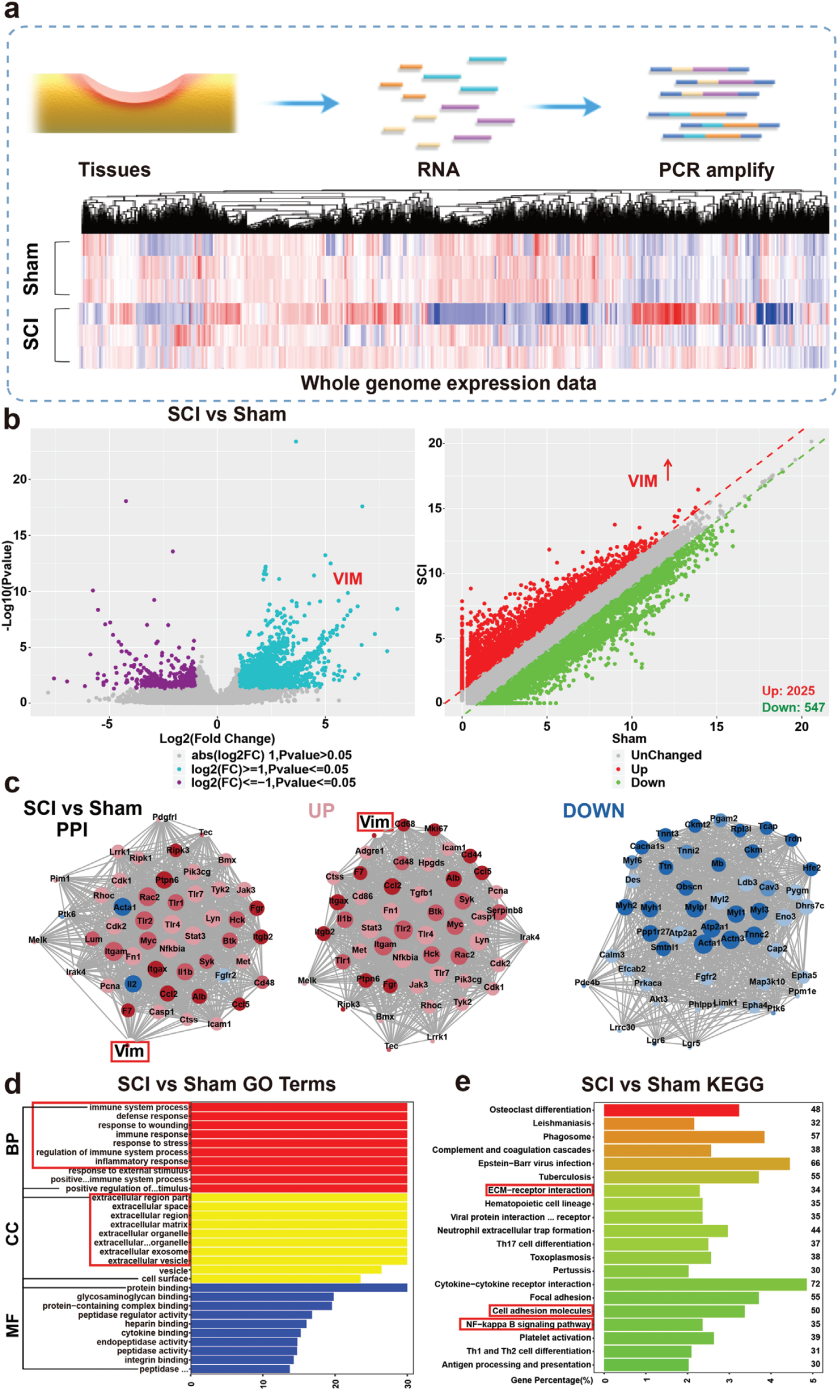
Transcriptome analysis of spinal cord compression injury in mice. a) Heat map analysis of whole expressed genes of the Sham group and SCI group. b) Volcano map and scatter plot analysis of Differentially Expressed Genes (DEGs) between the SCI group and Sham group. c) Protein–protein interaction network (PPI) analysis of the Sham group compared to the SCI group. Red represents upregulated proteins and blue represents down‐regulated proteins. d) Gene Ontology (GO) terms of the SCI group compared to the Sham group. e) Top 20 enriched Kyoto Encyclopedia of Genes and Genomes (KEGG) pathways up/downregulated in the SCI group compared to the Sham group (*N* = 3 in each group).

The study entailed the selection of spinal cord samples from C57 wild‐type male mice subjected to modeling using the Nystrom^[^ ^32^ ^]^ method over an 8‐week period. RNA sequencing was conducted on these samples, with each group comprising three replicates to facilitate comparative analysis. High‐throughput sequencing of both the SCI group and Sham group generated ≈ 50 million reads per sample (**Figure** **2**a). Differential expression analysis comparing the SCI group to the Sham group revealed a total of 2572 significantly differentially expressed genes (DEGs). Among them, 2025 genes exhibited upregulation in the 3D‐ISN group, while 547 genes showed downregulation (Figure 2b).

Through an integrated analysis of DEGs and protein–protein interaction (PPI), the pivotal role played by the Vimentin protein in the network was elucidated. Vimentin displayed binding interactions with numerous inflammation‐related proteins, including chemotactic factors, cytokines, and acute response proteins, underscoring its crucial involvement in inflammation regulation. Further upstream regulatory factor analysis indicated a significant upregulation of the *VIM* gene in astrocytes following neural damage, a phenomenon closely associated with the abnormal spatial expansion of astrocytes (Figure 2c).^[^ ^33^ ^]^

In the SCI, reactive astrocytes exhibited aberrant expansion properties, often accompanied by excessive expression of Vimentin, suggesting an intensified response of astrocytes in the injured area. This abnormal expansion likely involves significant morphological transformations, including increased cellular protrusions and branching, indicative of heightened astrocytic activity in the damaged environment. The over‐expression of Vimentin may play a pivotal role in this process, serving as an intermediate filament protein responsible for supporting the cellular cytoskeleton. Its aberrant expression may lead to abnormal morphological and structural transformations. The observation of this abnormal expansion behavior indicates the involvement of astrocytes in cellular activities beyond the normal repair requirements, potentially resulting in excessive tissue reaction or increased cell migration, thereby impacting the overall recovery of neural tissue. This observation provides insights into the abnormal functionality of astrocytes in the nervous system, paving the way for further in‐depth research into the mechanisms of neural injury and repair.

Additionally, Gene Ontology (GO) analysis was undertaken, with a specific focus on cellular components (CC), revealing a notable enrichment of DEGs in key targets associated with the extracellular matrix (ECM). This enrichment implies the potential significance of the ECM during SCI and repair processes (Figure 2d). Simultaneously, Kyoto Encyclopedia of Genes and Genomes (KEGG) enrichment analysis revealed an upregulation in genes related to “cell adhesion,” “ECM‐receptor interaction,” and the “NF‐κB signaling pathway” (Figure 2e). The upregulation of “cell adhesion” and “ECM‐receptor interaction” suggests an increase in cell migration in the injured area, with enhanced interactions between the ECM and receptors on the cell membrane.^[^ ^34^, ^35^ ^]^ This likely indicates that the reactive astrocytes during the injury exert pressure on the regenerative living space of normal cells, resulting in adverse effects. Therefore, it is hypothesized that by designing 3D structures of fibrous biomaterials to modulate the normal ECM, its spatial structure may influence the abnormal spatial expansion of astrocytes, thereby modulating Vimentin protein expression. This presents a novel strategy for the treatment of central nervous system injuries.

### 2.2 Characterization of 3D Electrospun Short Fibers

**Figure 3:**
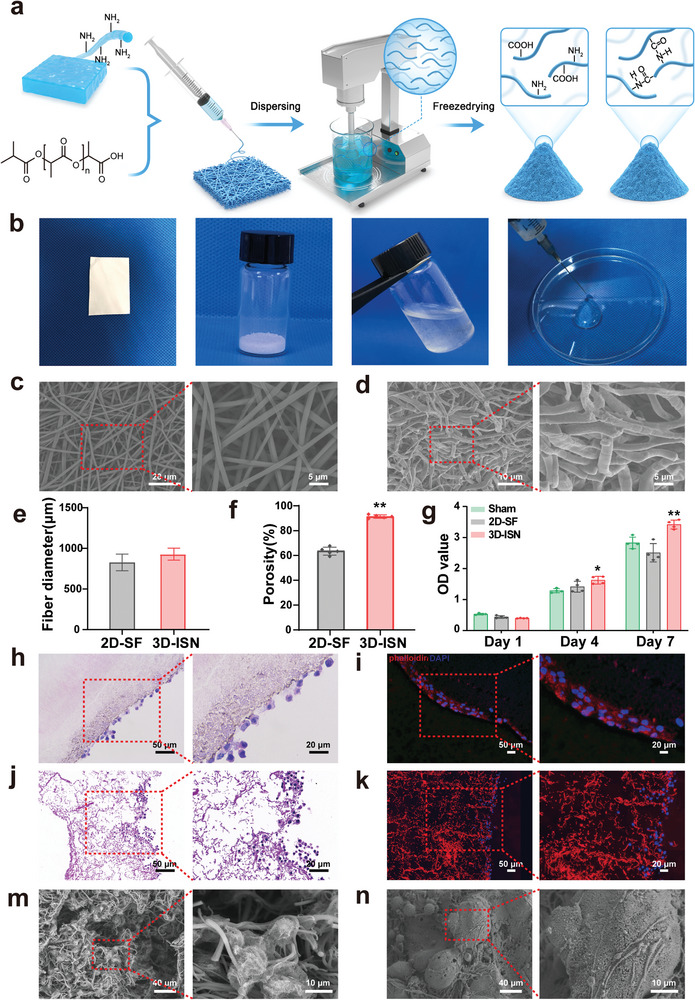
Preparation and Characterization of 3D electrospun short fibers. a) Schematic representation of the preparations with 2D‐SF and 3D‐ISN. b) Macroscopic images of 2D‐SF and 3D‐ISN. c,d) SEM of 2D‐SF and 3D‐ISN. e) Fiber diameters of 2D‐SF and 3D‐ISN. f) Porosity of 2D‐SF and 3D‐ISN. g) CCK8 results of neuroglia cells co‐cultured with 2D‐SF and 3D‐ISN at different time points. (Data presented as mean ± SEM, *N* = 3 in each group, *P*‐values are calculated using one‐way ANOVA, *p < 0.05, **p < 0.01.) h–k) Hematoxylin & Eosin (HE) staining and immunofluorescence staining after co‐culturing neuroglial cells with 2D‐SF and 3D‐ISN for 7 days. m,n) SEM images of neuroglial cells and neural stem cells within the 3D ISN.

Firstly, 2D fibrous membranes (2D‐SF) and 3D short fibers (3D‐ISN) were prepared according to the procedure outlined in **Figure** **3**a. The overall appearance of both fibers is shown in Figure 3b. The 2D‐SF represents the comprehensive structure of the nanofiber membrane, characterized by uniformity and flatness in surface smoothness at a macroscopic level. The homogeneous dispersion of 3D‐ISN in aqueous solutions underscores its stability and even distribution in solution. Additionally, 3D‐ISN can be administered via a 5 mL syringe, as depicted in Figure 3b, demonstrating its controllability in small‐volume devices and offering potential solutions for convenient medical applications.

Furthermore, the microscopic appearance and morphology of both 2D‐SF and 3D‐ISN were assessed using scanning electron microscopy (SEM) (Figure 3c,d). The 2D‐SF exhibited uniform distribution without evident stacking or crossing points, resulting in a relatively consistent fiber density across the surface. These fibers displayed a randomly oriented arrangement in a 2D plane, forming a tightly knit network structure, indicative of their uniform distribution. SEM analysis revealed a straightforward 2D planar structure for 2D‐SF, lacking any discernible 3D characteristics (Figure 3c). However, the 3D ISN appeared smooth without any discernible particles or imperfections. Upon high magnification, the microstructure of the fibers was clearly discernible, with a compact and uniform fibrous structure, highly similar to the natural ECM (Figure 3d). No significant difference in mean diameter between 2D‐SF and 3D ISN was found (Figure 3e). Notably, 3D ISN demonstrated a higher porosity of 96% compared to 2D‐SF (Figure 3f), indicating that 3D‐ISN has a unique 3D spatial structure. This internal 3D spatial network structure offers multiple dimensions for cell attachment and growth regulation.

### 2.3 In Vitro Characterization of Neuroglial Cells with 3D‐ISN

Neuroglial cells were co‐cultured with 2D‐SF and 3D‐ISN to investigate the effects of these two different spatial types of nanofibers on cells and organisms, including cell attachment, proliferation, and differentiation. The proliferation of neuroglial cells was quantitatively assessed using CCK‐8 assays. The analysis revealed that compared to the control and 2D‐SF groups, the absorbance of 3D‐ISN was significantly higher on day 7, indicating enhanced cell proliferation activity. Conversely, the 2D‐SF group exhibited no significant effect on cell proliferation compared to the control group (Figure 3g). Hematoxylin & Eosin (HE) staining and fluorescence staining of the cytoskeleton and nuclei of the nanofibers after 7 days of co‐culture showed that 2D‐SF provided limited space, with neuroglial cells mainly attaching to the surface of the nanofibers and growing on their surface (Figure 3h,i). In this scenario, cell growth was primarily constrained by the structure of the fibers. Moreover, the confined 3D space offered by 2D‐SF significantly impeded cell penetration into the fiber interior. Conversely, in 3D‐ISN, neuroglial cells could fully establish 3D structures, indicating that the 3D environment affords a more expansive space for cellular interaction and growth (Figure 3j,k). Consequently, neuroglial cells proliferated and expanded in multiple directions, forming intricate 3D structures. Additionally, the 3D environment likely enhances cell–fiber interactions, enabling neuroglial cells to grow more freely along the surface and interior of fibers. These findings suggest that 3D‐ISN possesses good biocompatibility, and its spatial morphology may be more conducive to regulating the proliferation microenvironment of neuroglilal cells.

To gain deeper insights into the microscopic morphology of neuroglial cell and primary mouse neural stem cells (NSCs) growth on the 3D‐ISN fibers, cell SEM analysis was conducted (Figure 3m,n). Neuroglial cells and NSCs on the fibers demonstrated morphological flattening and extension, suggesting that 3D‐ISN provides an effective scaffold for nerve cells interact and migrate with fibers.

### 2.4 The Effects of 3D‐ISN on the Behavior of Mice after SCI

**Figure 4:**
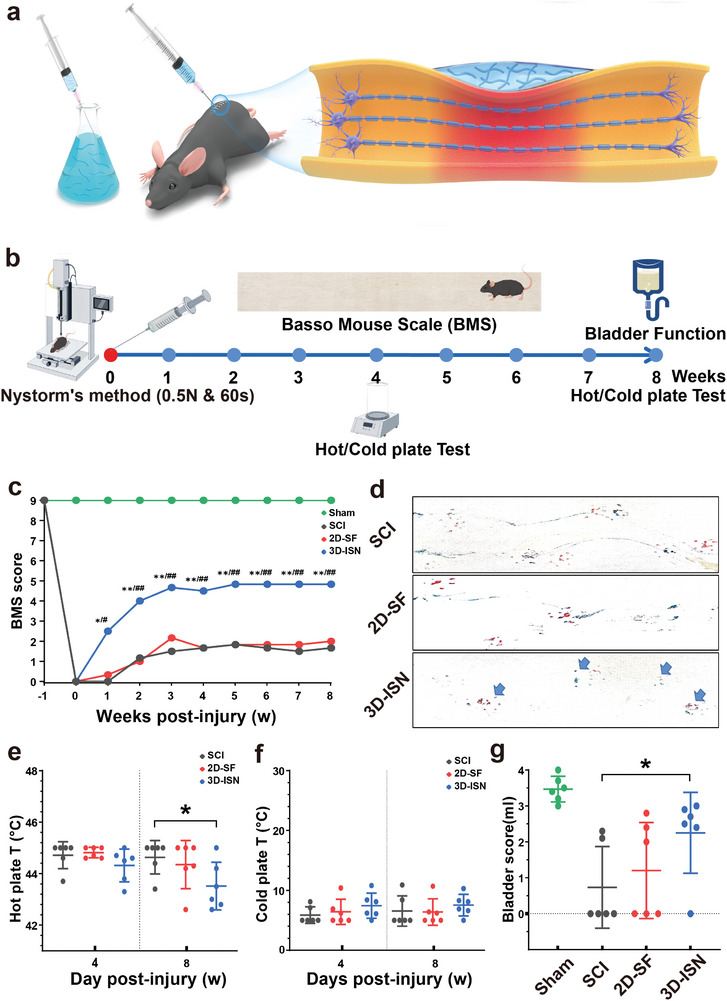
The effects of 3D‐ISN on the behavior of mice after SCI. a) Schematic diagram of spinal cord compression injury in mice following orthotopic 3D‐ISN injection. b) Schematic diagram of the mouse model of spinal cord compression injury and the time point of behavioral scores. c) BMS score of mice with SCI after 3D‐ISN injection. d) Behavioral footprint test (56 days after injury; blue arrows point to hind limb fingerprints. e) Cold plate test at 4 and 8 weeks after SCI. f) Hot plate experiments in mice 4 and 8 weeks after injury. g) Bladder function scores (residual urine volume analysis) in mice 8 weeks after injury. (*N* = 6 in each group, *p*‐values are calculated using one‐way ANOVA, ^*/#^ *p* < 0.05, ^**/##^ *p *< 0.01).

Wild‐type C57 male mice underwent treatment with 2D‐SF implantation and 3D‐ISN injection at lesion sites on the day following SCI modeling (**Figure** **4**a). It is worth emphasizing that, whether implanted or injected, an equal mass of both 2D‐SF and 3D‐ISN was administered. Subsequently, the mice were assessed for motor recovery using a series of scores (Figure 4b). As depicted in Figure 4c,d, all mice displayed severe motor deficits in the Basso Mouse Scale (BMS)^[^ ^36^ ^]^ during the initial postoperative phase, indicating the successful establishment of the SCI model. Notably, mice treated with 3D‐ISN exhibited significant improvement in motor function. The BMS scores of the 3D‐ISN group were markedly higher than those of the SCI and 2D‐SF groups (*p* < 0.01), underscoring the efficacy of 3D‐ISN treatment in promoting motor function recovery. Following 3D‐ISN treatment, substantial recovery of sensory function was observed in the hot plate test. The response time of these mice was notably shorter compared to mice treated with 2D‐SF, indicating the effectiveness of 3D‐ISN treatment in facilitating heat sensory function recovery in mice. However, it had no apparent effect on cold sensation (Figure 4e,f). Additionally, a favorable impact of 3D‐ISN treatment on bladder function recovery in mice was noted. The bladder storage function of these mice approached a more normal range compared to the 2D‐SF group, suggesting the efficacy of 3D‐ISN in promoting bladder nerve function recovery (Figure 4g).

Taken together, these findings indicate a role for 3D‐ISN in promoting motor recovery following SCI. While these results are promising, further investigations are warranted to elucidate the underlying mechanism of action. Such endeavors will contribute to a comprehensive understanding of the potential benefits and application prospects of 3D‐ISN, thus providing valuable insights for guiding future research and clinical practice.

### 2.5 Sagittal HE Staining and Neuronal Fluorescence Staining of SCI

**Figure 5:**
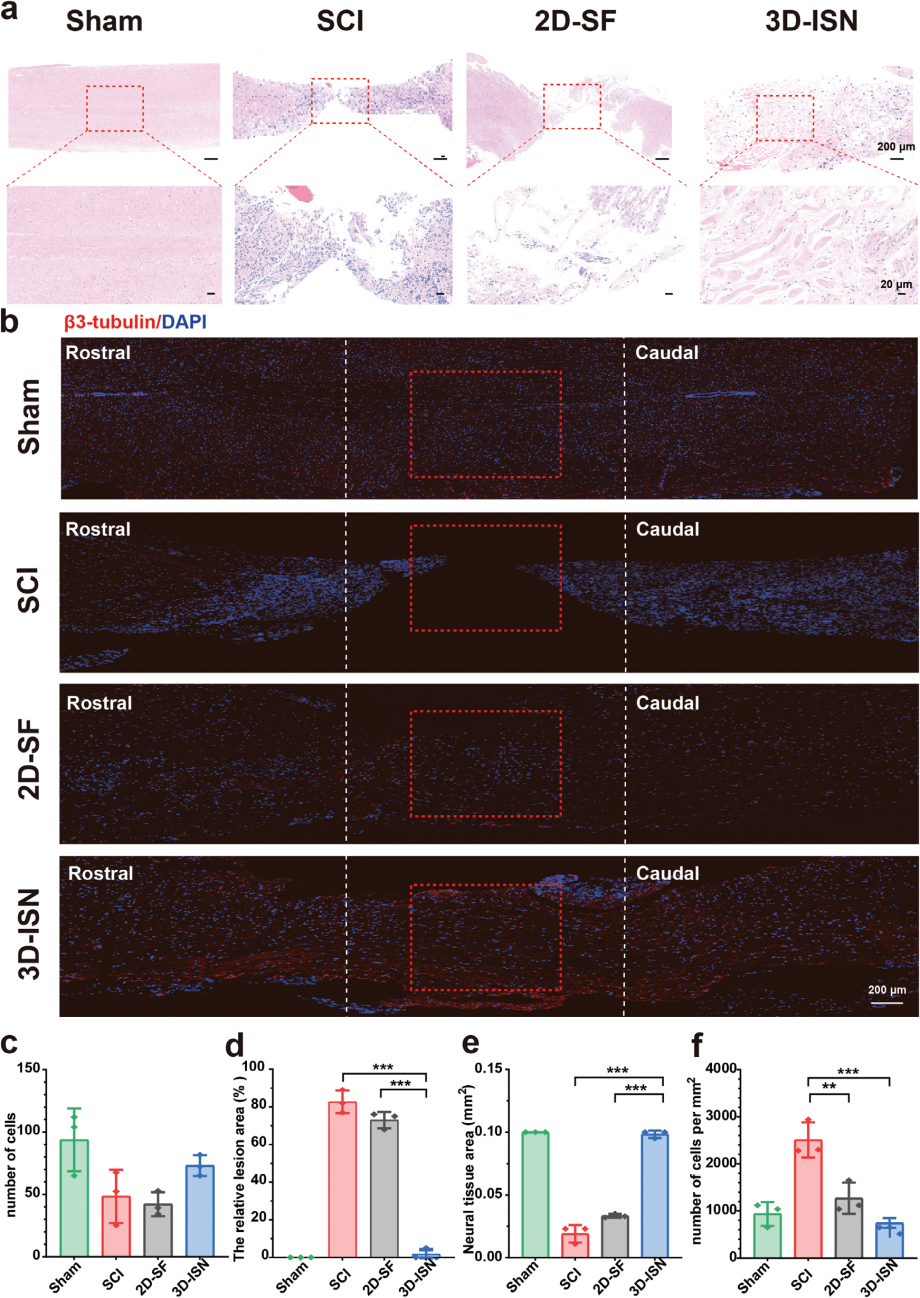
Sagittal HE staining and neuronal fluorescence staining of SCI. a) Sagittal HE stained image of the spinal cord. b) Immunofluorescence staining of spinal cord neurons with β3‐tubulin‐stained cells (red); nuclei were stained with 4, 6‐diamino‐2‐phenylino (DAPI) (blue). c) The number of nuclei at the injury site. d) The proportion of defective tissue at the injury site. e) The area of nerve tissue at the injury site. f) Quantitative analysis of the number of cells per unit area. (Data presented as mean ± SEM, *N* = 3 in each group, *p*‐values are calculated using one‐way ANOVA, **p < 0.01, ***p < 0.001).

Following 3D‐ISN treatment, the spinal cord underwent sectioning, and HE staining was conducted to assess morphological changes in the neural tissue. HE staining revealed significant neural tissue recovery in the injured area of the spinal cord in the 3D‐ISN group compared to the 2D‐SF group. In the SCI group, HE staining depicted pronounced cell death, inflammation, tissue defects, and cavity formation (**Figure** **5**a,d,e). However, post 3D‐ISN treatment, inflammation at the injury site significantly decreased, nerve cell survival notably improved, and better tissue integrity was observed in the SCI group. Notably, 3D‐ISN effectively regulated cell numbers post‐SCI, approaching levels comparable to the normal spinal cord when HE‐stained cells were quantified in each group (Figure 5c,f).

During SCI, nerve cell death typically occurs due to direct mechanical damage, ischemia, oxidative stress, excitotoxicity, inflammatory response, and apoptosis. However, increased cell numbers during the later stages of SCI can be attributed to two main factors. First, the immune system initiates an inflammatory response post‐SCI, leading to an accumulation of immune cells (such as macrophages and lymphocytes) at the injury site, potentially increasing the total cell count.^[^ ^37^, ^38^ ^]^ Additionally, reactive astrocytes rapidly expand in an abnormal manner to form scar tissue, contributing to elevated cell numbers. Thus, if 3D‐ISN can maintain normal cell numbers in nerves, it may serve to protect neural tissue, mitigate inflammation, and suppress scar formation.^[^ ^39^ ^]^ Moreover, these findings suggest that the efficacy of 3D‐ISN treatment may extend beyond initial protection, potentially persisting over time post‐injury by enhancing the microenvironment and supporting nerve cell survival and regeneration.

Furthermore, longitudinal sections of the spinal cord underwent full‐length staining to assess spinal cord recovery post‐injury (Figure 5b). It is plausible that 3D‐ISN may enhance the number of β3‐tubulin^+^ cells by providing a conducive microenvironment, thus promoting the differentiation of neural progenitor cells into neurons or safeguarding existing neurons from damage. This positive outcome suggests that 3D‐ISN could contribute to neurological function recovery following SCI. The spatial structure of 3D‐ISN is hypothesized to underlie these effects, although further experiments, including the use of more specific cell markers and activity tests, are required to substantiate this hypothesis and elucidate the mechanistic actions and effects of 3D‐ISN.

### 2.6 Cell Labeling and Immunofluorescence Evaluation

**Figure 6:**
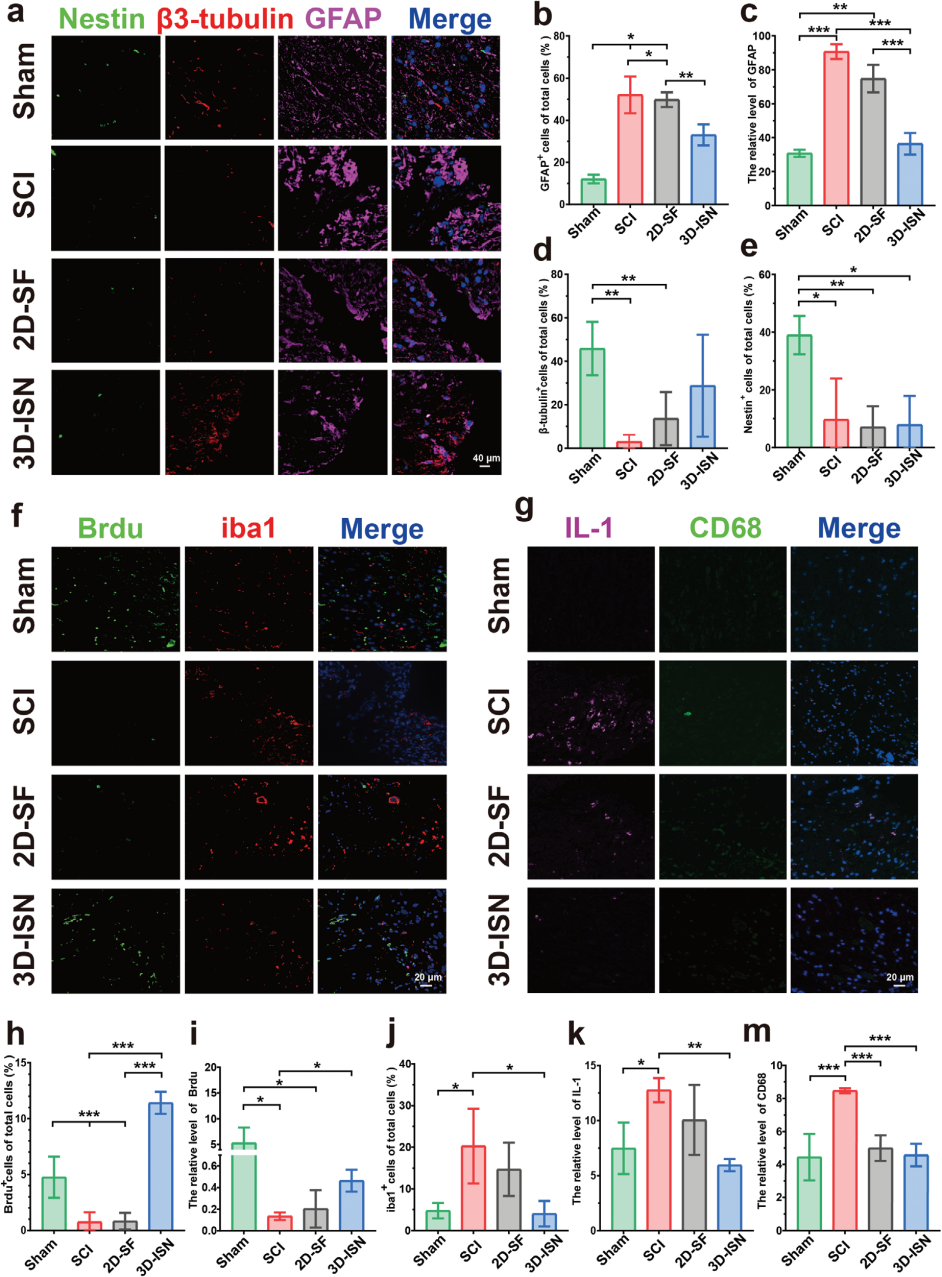
Cell labeling and immunofluorescence evaluation. a) Immunofluorescence image analysis of different cell types following SCI in the Sham group, SCI group, 2D‐SF group, and 3D‐ISN group, including Nestin (green), β3‐tubulin (red), and GFAP (pink); nuclei were stained with 4, 6‐diamino‐2‐phenylino (DAPI) (blue). b) The proportion of GFAP‐positive cells. c) Relative fluorescence intensity of GFAP. d) The proportion of β3‐tubulin‐positive cells. e) The proportion of nestin‐positive cells. f) Immunofluorescence staining images of SCI showing cells stained with BrdU (green), Iba1 (red), and nuclei stained with 4, 6‐diamino‐2‐phenyline (DAPI) (blue). g) Immunofluorescence staining images of inflammatory factors at the site of SCI, showing cells stained with iL‐1 (pink), CD68 (green), and nuclei stained with 4, 6‐diamino‐2‐phenylino (DAPI) (blue). h) The proportion of BrdU‐positive cells. i) The relative fluorescence intensity of BrdU. j) The Proportion of Iba1‐positive cells. k) The relative fluorescence intensity of iL‐1. m) The relative fluorescence intensity of CD‐68. Data are presented as mean ± SD of at least three independent experiments and were analyzed by one‐way ANOVA with Tukey's post hoc multiple comparison test. (Data presented as mean ± SEM, *N* = 3 in each group, *p*‐values are calculated using one‐way ANOVA, *p < 0.05, **p < 0.01, ***p < 0.001).

Immunofluorescence staining of Nestin^+^, GFAP^+^, and β3‐tubulin^+^ cells, along with their expression in spinal cord tissues, provided significant insights into the mechanistic actions and effects of 3D‐ISN on neurons. Nestin serves as a specific marker for neural stem cells and neural precursor cells. It was observed that the proportion of Nestin^+^ cells decreased in each group after injury, suggesting migration and differentiation of stem cells into more mature neural cell types, such as neurons or neuroglia cells, to facilitate neural tissue repair (**Figure** **6**a,e).

An especially notable observation was the observation of GFAP astrocyte‐specific labeling (Figure 6a–c). Elevated GFAP expression levels are indicative of reactive astrogliosis. In this study, a significant increase in the number of GFAP^+^ astrocytes was noted in the SCI group, accompanied by a sharp rise in GFAP protein expression levels and area (*p* < 0.01), indicating pronounced abnormal expansion of astrocytes post‐injury. In contrast, the proportion of GFAP^+^ cells significantly decreased in the 3D‐ISN group, with no notable difference in the number of GFAP^+^ cells compared to the Sham group. Consistently, the relative protein content of fluorescently labeled GFAP mirrored these trends. Overall, these findings suggest that 3D‐ISN may constrain the spatial expansion of astrocytes and maintain normal astrocyte levels by inhibiting reactive astrogliosis post‐injury. The reactive astrocytes may trigger the release of inflammatory factors, exacerbating the inflammatory response in the nervous system and negatively impacting normal neurological function. By reducing the number of GFAP^+^ cells, 3D‐ISN could create a more conducive environment for neuronal regeneration, thereby aiding in the recovery of neurological function post‐SCI.

Furthermore, a significant increase in the proportion of β3‐tubulin^+^ cells, a specific marker for mature neurons, was observed in the 3D‐ISN group compared to the 2D‐SF and SCI groups, suggesting enhanced neuronal generation by 3D‐ISN (Figure 6a,d). Neurons play a pivotal role in transmitting and processing information, and their loss following SCI contributes to dysfunction. Thus, promoting neuronal generation or protecting the survival of existing neurons is a crucial treatment of SCI. Moreover, BrdU staining indicated a significant increase in the proportion of BrdU^+^ cells and fluorescence intensity in the 3D‐ISN group compared to other groups (Figure 6f,h,i). BrdU, a synthetic nucleoside, labels proliferating cells, suggesting that 3D‐ISN may support neuronal regeneration by promoting the generation of neuronal cells and creating a favorable environment.

Subsequently, the investigation delved into the level of secondary inflammation. Iba1 serves as a well‐known marker of microglial activation, crucial in inflammation and wound healing within the central nervous system. Following SCI, the proportion of Iba1^+^ microglial cells increased due to the neuroinflammatory response (Figure 6f,j). Notably, the reduction in Iba1 expression post‐injection of short fibers in the experimental 3D‐ISN group suggested the effective attenuation of the neuroinflammatory response and suppression of microglial activation, both advantageous for nerve injury repair. CD68, a highly glycosylated transmembrane protein, is widely expressed in monocyte‐macrophage cell lines across various tissues and is often utilized as a neuroinflammation marker within the central nervous system. IL‐1 (interleukin‐1), composed of IL‐1α and IL‐1β forms, plays a pivotal role in numerous inflammatory and immune responses, predominantly produced by activated macrophages. During inflammatory conditions like nerve injury, disease, or infection, an increase in CD68^+^ neuroglia cells is observed, secreting IL‐1 and other inflammatory mediators, thereby exacerbating the inflammatory response (Figure 6g,k,m). Consequently, the expression levels of CD68 and IL‐1 serve as indicators to assess neuroinflammation severity. The reduction in CD68^+^ cells and IL‐1 fluorescence intensity within the 3D‐ISN group may signify diminished inflammatory response and mitigation of nerve tissue damage.

Overall, these findings indicate the effective inhibition of reactive astrocyte expansion, reduction in inflammation levels, and maintenance of a conducive microenvironment for astrocyte generation by 3D‐ISN. While these experiments shed light on therapeutic effects, molecular mechanisms underlying these events, including key genes and specific signaling pathways, remain largely unknown. To address these unresolved queries, in‐depth analyses utilizing bioinformatics techniques are planned to elucidate therapeutic mechanisms comprehensively. This endeavor will foster a deeper understanding and pave the way for future research and therapeutic regimen development.

### 2.7 Whole Transcriptome Analysis

**Figure 7:**
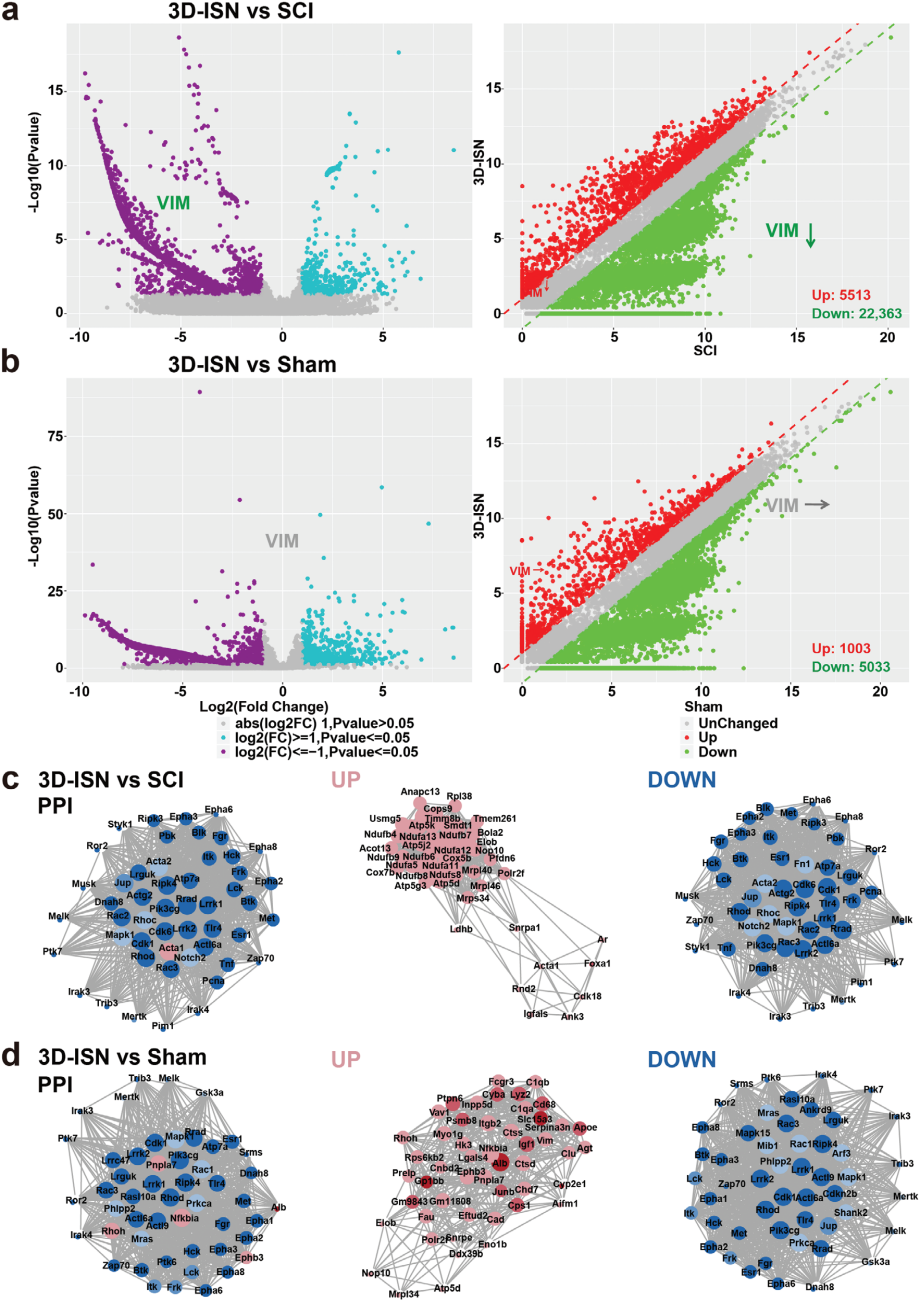
Whole transcriptome analysis of the Sham group, SCI group, and 3D‐ISN group. a) Volcano map and scatter plot analysis of DEGs between the 3D‐ISN group and SCI group. b) Volcano map and scatter plot analysis of DEGs between the 3D‐ISN group and Sham group. c) PPI analysis of the 3D‐ISN group compared to the SCI group. d) PPI analysis of the 3D‐ISN group compared to the Sham group. (Red represents up‐regulated proteins and blue represents down‐regulated proteins, *N* = 3 in each group).

In this study, RNA sequencing was conducted on treated spinal cord samples from the Sham, SCI, and 3D‐ISN groups, each comprising three replicates, to compare the results among the groups. High‐throughput sequencing generated ≈50 million reads per sample. The differential expression analysis revealed 27 876 significantly differentially expressed genes (DEGs) in the 3D‐ISN group, with 5513 genes upregulated and 22 363 genes downregulated compared to the SCI group (**Figure** **7**a).

First, upregulation of certain genes crucial for neural repair was observed following 3D‐ISN treatment, supporting previous findings that 3D‐ISN could facilitate neuronal growth and regeneration. Second, significant downregulation of genes related to the inflammatory response was noted after 3D‐ISN injection, suggesting that 3D fibers might mitigate the inflammatory response post‐nerve injury, representing a critical aspect of nerve repair. Notably, the expression level of the *VIM* gene decreased post‐3D‐ISN treatment, remaining relatively stable compared to the sham group (Figure 7a,b). This reduction may suggest the effectiveness of 3D‐ISN in inhibiting reactive astrogliosis commonly seen in nerve injuries, thereby facilitating nerve repair. Thus, 3D‐ISN could play a significant role in the SCI repair process by suppressing reactive astrogliosis and regulating *VIM* expression. However, further investigations are warranted to ascertain the direct relationship between reduced *VIM* expression and the therapeutic efficacy of 3D‐ISN, as well as to elucidate the underlying regulatory mechanisms, thereby enhancing the understanding of 3D fiber involvement in nerve repair and its therapeutic potential. Moreover, the PPI analysis revealed no significant interaction network involving the Vimtine protein (Figure 7c,d).

**Figure 8:**
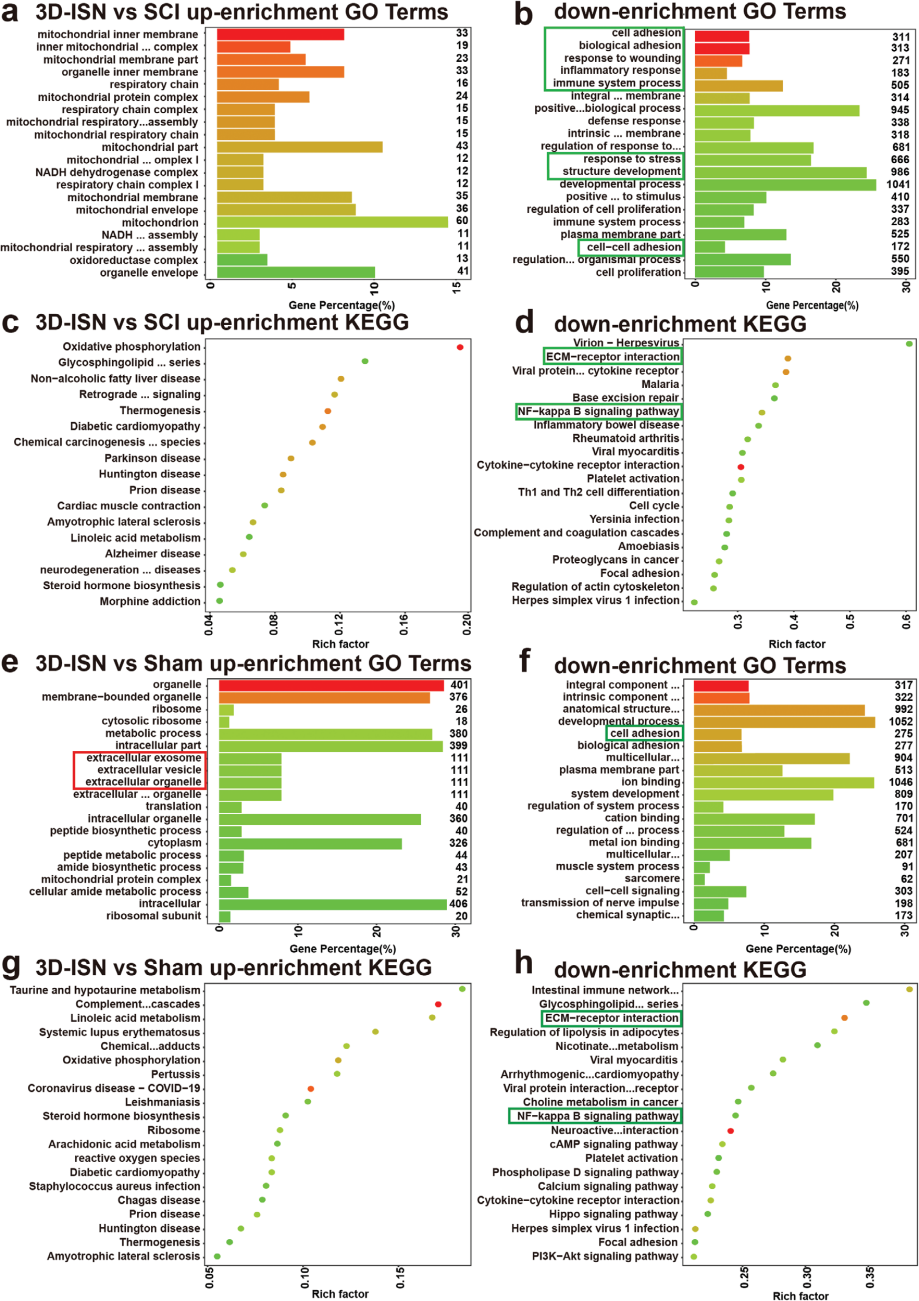
GO and KEGG enrichment analysis of the Sham group, SCI group, and 3D‐ISN group. a,b) The top 20 up/down‐regulated GO terms of the 3D‐ISN group compared to the SCI group. c,d) The top 20 up/down‐regulated KEGG pathways of the 3D‐ISN group compared to the SCI group. e,f) The top 20 up/downregulated KEGG pathways of the 3D‐ISN group compared to the Sham group. g,h) The top 20 up/downregulated KEGG pathways of the 3D‐ISN group compared to the Sham group (*N* = 3 in each group).

GO and KEGG enrichment analyses were employed to predict gene and molecular interaction networks, uncovering various biological processes, including signal transduction, disease occurrence, and metabolic pathways (**Figure** **8**a–h). It was hypothesized that Vimentin inhibition by 3D‐ISN could further suppress the NF‐κB pathway and confer protection against nerve injury (Figure 8b,d,f,h). NF‐κB is a transcription factor pivotal in immune response, cell growth, and apoptosis. Activation of the NF‐κB pathway is triggered by inflammation, external stimuli, or cellular stress, culminating in the binding of NF‐κB to DNA and subsequent modulation of gene expression. According to the transcriptome analysis results, 3D‐ISN effectively inhibited NF‐κB pathway activation (Figure 8d,h). This finding is significant as NF‐κB pathway activation is closely linked to neuroinflammation and neuronal demise, major impediments to neurological recovery post‐SCI. This finding is significant as NF‐κB pathway activation is closely linked to neuroinflammation and neuronal demise, major impediments to neurological recovery post‐SCI. Hence, attenuation of the neuroinflammatory response by 3D‐ISN might foster neurological function recovery by dampening NF‐κB pathway activation and inhibiting neuronal apoptosis.

Nevertheless, further investigations are imperative to validate these findings, explore the role of 3D‐ISN in maintaining astrocyte spatial homeostasis and suppressing NF‐κB pathway activation, and assess the contribution of these mechanisms to SCI repair. Such insights would deepen our understanding of how 3D‐ISN could enhance neurological recovery post‐SCI by modulating key molecular pathways. The potential of 3D‐ISN to influence multiple aspects of cellular behavior underlines the importance of comprehensive studies to fully elucidate its mechanisms of action.

### 2.8 Single‐Cell Sequencing Analysis in Astrocytes

**Figure 9:**
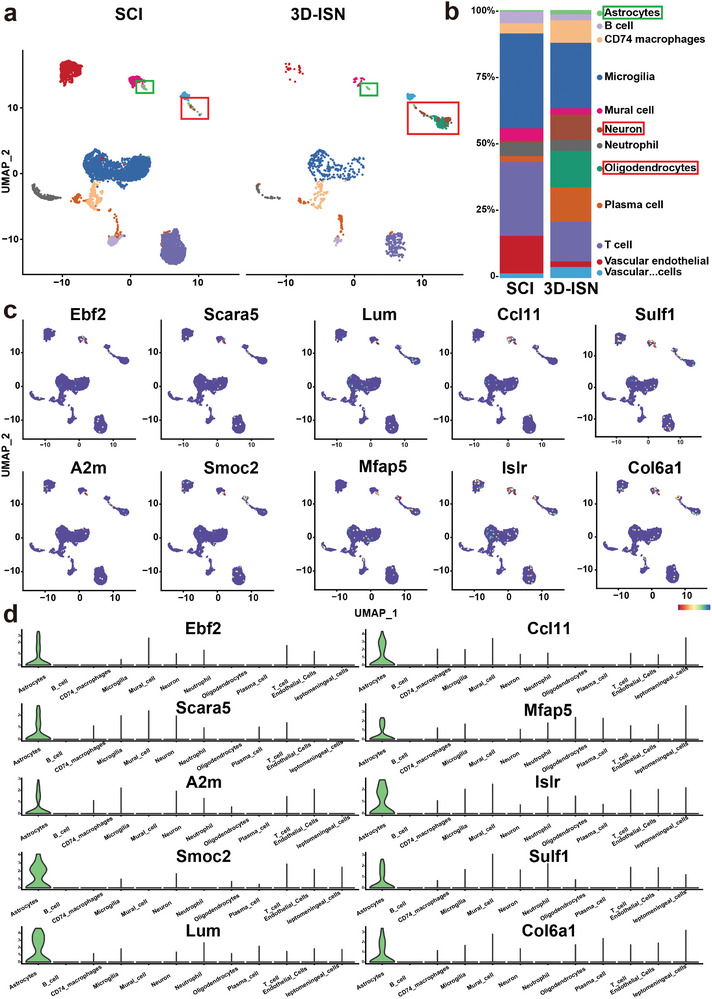
Cell clustering and cell identification by single‐cell sequencing: a) UMAP showed that cells in the spinal cord tissue existed in 12 distinct clusters. b) The proportion of cells in each cluster in the SCI group and the 3D‐ISN group. c) The expression of the most important unique genes in astrocytes and their UMAP maps. d) Violin plot of the unique genes in astrocytes (*N* = 3 in each group).

Subsequent to conducting single‐cell sequencing, the spinal cord tissue samples from both the SCI and 3D‐ISN groups were analyzed, revealing a total of 12 distinct cell subsets, including neurons, astrocytes, and microglia (**Figure** **9**a). After Quality Control (QC), there were 6886 cells in the SCI group and 1061 cells in the 3D‐ISN group. In the SCI group, activated microglia were the predominant cell type, comprising 35.3% of the total cells (2301 cells), whereas in the 3D‐ISN group, microglia accounted for 24.4% (257 cells) (Figure 9b). The SCI group contained 40 neurons (0.6%), whereas the 3D‐ISN group had 98 neurons (9.3%). Additionally, by utilizing specific genes as markers for astrocytes (Figure 9c,d), it was observed that there were 17 astrocytes in the 3D‐ISN group, significantly fewer than the 41 astrocytes in the SCI group. These single‐cell clustering findings were consistent with previous immunofluorescence results, indicating that 3D‐ISN can mitigate reactive astrogliosis, optimize cellular composition, and facilitate neuron regeneration.

**Figure 10:**
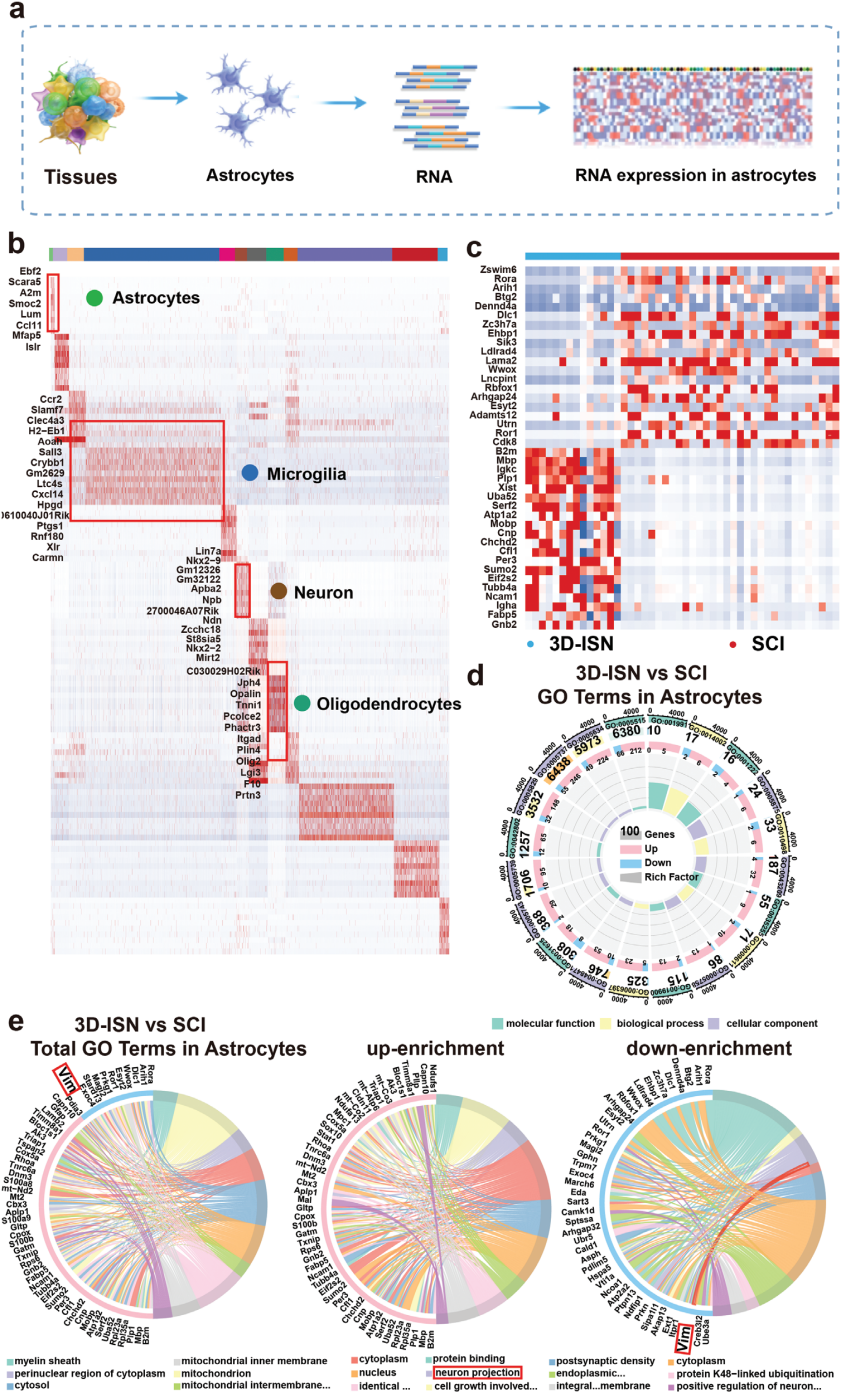
Single‐cell sequencing analysis in astrocytes: a) Schematic diagram of single‐cell sequencing of astrocytes. b) Heat map analysis of marker gene expression in the main cell clusters. c) Heat map analysis of DEGs of the 3D‐ISN group compared to the SCI group in astrocytes from single‐cell sequencing. d,e) GO plot of Up/downregulated genes expressed of the 3D‐ISN group compared to the SCI group in astrocytes (*N* = 3 in each group).

Subsequently, marker genes expressed by different cell types were identified (**Figure** **10**a,b), and DEGs in astrocytes from both groups were analyzed (Figure 10c). Notably, consistent with whole transcriptome RNA‐sequencing results, although the *VIM* gene was not among the top 20 DEGs, its expression was significantly downregulated in the astrocytes of the 3D‐ISN group. This indicates alterations in astrocyte cytoskeleton structure and migration capacity that underscore the effect of 3D‐ISN on cellular behavior critical for neural repair.

**Figure 11:**
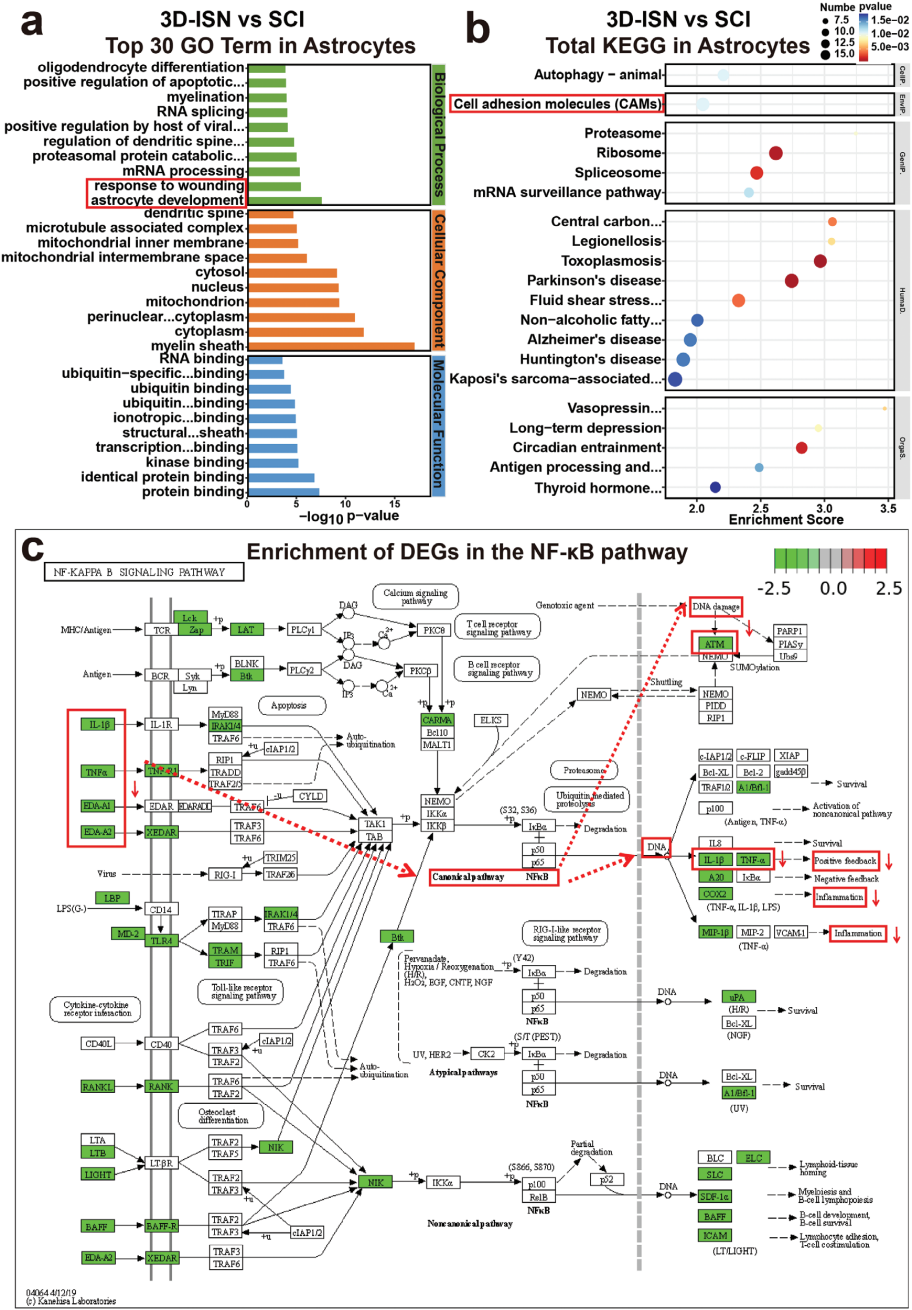
GO and KEGG enrichment analysis of the 3D‐ISN group in astrocytes: a) Up/downregulated GO terms of the 3D‐ISN group compared to the SCI group. b) KEGG enrichment chart of the 3D‐ISN group compared to the SCI group. c) The enrichment of DEGs between the 3D‐ISN group and the SCI group in the NF‐κB signaling pathway (*N* = 3 in each group).

Furthermore, GO analysis revealed that compared to the SCI group, the 3D‐ISN group exhibited downregulation of genes related to “Astrocyte development,” as well as genes associated with “regulation of cell adhesion” and “positive regulation of neuron projection” (Figure 10d,e, and **11**a). These findings indicate that while restraining reactive astrogliosis, there is also a beneficial modulation of neuronal synapse formation. This dual action of 3D‐ISN highlights its potential to both limit detrimental cellular responses and promote positive outcomes in neural tissue repair. Additionally, KEGG enrichment analysis found significant downregulation of “cell adhesion.” These results suggest that 3D‐ISN, through its unique 3D confined space structure, inhibits cell adhesion, leading to suppression of reactive astrocytes and ultimately weakening the process of “Astrocyte development” (Figure 11b).

These single‐cell sequencing results suggest that 3D‐ISN may reduce the reactive astrocytes by regulating fibrous‐cellular interactions. Combined with whole transcriptome analysis, it is speculated that the downregulation of *VIM* gene expression could result in decreased Vimentin protein levels. Vimentin, an intermediate filament protein involved in cell spatial expansion, interacts with inflammation‐related proteins in central nervous system injury. Reduction in *VIM* gene expression may decrease Vimentin protein levels, potentially disrupting its interaction with inflammatory proteins (including TNF‐α and IL‐1β) (Figure 11c). This disruption could affect various signal transduction events, including NF‐κB pathway activity, crucial for regulating inflammatory responses, cell proliferation, and survival.

In summary, the above single‐cell sequencing results elucidate the regulatory effects of 3D‐ISN on astrocytes. The presence of 3D‐ISN modulates cell adhesion, confines abnormal astrocyte expansion, slows down *VIM* gene expression, inhibits reactive astrogliosis, and ultimately promotes the regeneration and repair of neuronal synapses (Figures S1–S4, Supporting Information). These findings underscore the therapeutic potential of 3D‐ISN in spinal cord injury repair, providing a foundation for further research and development of effective treatments.

## 3 Conclusion

In this study, based on a whole transcriptome analysis of SCI, the primary matrix and targets of action for reactive astrogliosis were focused on. The 3D‐ISN was designed to constrain the abnormal spatial expansion of astrocytes. Through in situ injection into the injury site, its unique spatial structure was able to block “ECM receptor interactions,” effectively inhibiting the expression of the *VIM* gene in astrocytes and thereby preventing the transcription of downstream Vimentin protein. Moreover, by blocking the binding of Vimentin protein with TNF‐α and IL‐1β, the injectable system ultimately counter‐regulated the NF‐κB pathway, reducing the release of inflammatory factors and effectively suppressing neuron apoptosis. In this study, by regulating the spatial structure of fibers, the mechanism chain by which 3D fibers with spatial constraints promote the recovery of spinal cord nerve function was analyzed for the first time, showing potential for clinical application.

## 4 Experimental Section

### Materials

Gelatin sourced from pig skin was obtained from Sigma–Aldrich (St. Louis, United States). Poly‐lactic‐acid (PLA) was provided by Jinan Daigang Biomaterial Co., Ltd. (Jinan, China), and 1,1,3,3,3‐hexafluoro‐2‐propanol (HFIP) was supplied by Aladdin Industrial Co., Ltd. (Shanghai, China). Tert‐butanol alcohol was obtained from Rhawn Reagent Co., Ltd. (Guangzhou, China). Neural stem cells and neuroglial cells were extracted from the brain tissues of newborn mice following an ethical review. Cell culture reagents for neuronal cells were sourced from Thermo Fisher Scientific Co., Ltd. (Massachusetts, USA).

### Preparation and Characterization of 3D‐ISN

Injectable short fibers were prepared from gelatin and PLA by dissolving them in HFIP at a mass ratio of 4:1, resulting in a gelatin/PLA solution (12 wt.%). Electrospinning was conducted under optimal conditions: an applied voltage of 15 kV, a flow rate of 3 mL h^−1^, and a collection distance of 15 cm. The resulting gelatin/PLA nanofiber membranes were vacuum‐dried to remove residual solvents. The preparation of injectable short nanofibers involved three main steps: 1) cutting the fiber film into small fragments; 2) dispersing the fragments in tert‐butanol alcohol using the IKA homogenization method, followed by homogenization at 10 000 rpm for 15 min; 3) freezing in liquid nitrogen for 1 h, followed by freeze‐drying for 48 h to obtain non‐crosslinked short fibers. To stabilize the structure, the fibers were thermally crosslinked at 180 °C for 2 h. Subsequently, the crosslinked fibers were mixed with an aqueous solution and re‐homogenized at 10 000 rpm for 5 min to finally form injectable short fibers with a 2% mass concentration (3D‐ISN).

### Proliferation and Infiltration of Nerve Cells on 3D‐ISN

The fibrous scaffolds used to culture the cells were formed by naturally drying the 3D short‐fiber injectable solution. Neural stem cells or neuroglial cells were cultured on 2D‐SF and 3D‐ISN to study their behavior. Separate culture media for neurons and neuroglial cells were used.

Cell proliferation was assessed using the Cell Counting Kit‐8 (CCK8) assay. After co‐culturing neuroglial cells with the nanofiber scaffold for the specified period, the CCK8 solution (Dojindo Molecular Technologies, Inc.) was added to each well at a 1:10 dilution with the culture medium. The cells were then incubated at 37 °C for 2 h. The absorbance was measured at 450 nm using a microplate reader (Bio‐Rad Laboratories). The resulting absorbance values corresponded to the number of viable cells, indicating the proliferation rate.

HE staining, immunofluorescence staining and cell SEM were employed to examine neuroglial cell adhesion and infiltration within the scaffolds.

### Animal Models of SCI and Treatment

This study utilized 24 3‐month‐old C57 mice (≈25 g) as animal models to evaluate the therapeutic effects of injectable nanofibers on functional recovery after SCI. The mice were fed and experiments were carried out under protocols approved by the Animal Ethics Committee of Shanghai Jiao Tong University School of Medicine (Number: SYXK2018‐0027). The mice were randomly divided into four groups: Sham (*n* = 6), SCI (*n* = 6), 2D‐SF (*n* = 6), and 3D‐ISN (*n* = 6). Nystrom's method was employed to create the model. Animals were anesthetized with 10% (w/v) chloral hydrate (15 mL kg^−1^, i.p.), and after skin disinfection with 1% povidone–iodine, laminectomy was performed at the level of T10‐T12 to expose the thoracic vertebrae. Pressure was then applied to the spinal cord at T11 for 60 s with a weight twice that of the mouse. In the 2D‐SF group, the 2D fiber membrane was implanted and the wound was sutured layer‐by‐layer. The 2D‐SF used in this study was sufficient to cover the spinal cord injury site, with an approximate size of 0.1 × 0.1 cm. The mass is ≈ 0.2 mg. In the 3D‐ISN group, 0.1 mL of injectable short fibers were injected into T11 with a 1 mL syringe immediately after the nerve defect. Injectable short fibers at this volume had the same quality as 2D‐SF to ensure a fair comparison of their effects. This approach could directly compare the impact of different spatial structures on spinal cord repair.

### The Evaluation of Functional Behavior

2 weeks before the study began, mice were acclimatized to the open field to alleviate anxiety and pain. On the day of the behavioral tests, two researchers evaluated the hindlimb function and locomotion of the mice using the BMS without any treatment. Each mouse underwent testing on an incremental hot/cold plate, with temperatures gradually adjusted until injury‐related behavior was exhibited. The threshold temperature was recorded, and subsequent testing was performed at 30 and 60 days post‐injury using the Incremental Thermal Plate (ITP). For bladder function assessment, mean bladder scores ranging from 0 to 3 were calculated based on two daily measurements of urinary retention. A score of 0 indicated an empty bladder, 1 indicated a small bladder, 2 indicated a medium bladder, and 3 indicated a large & full bladder. The timing of bladder emptying and the individual responsible for facilitating the emptying were consistent throughout the experiment.

### Cell Labeling and Immunofluorescence Evaluation

Tissues were processed for immunofluorescence at 8 weeks post‐injury. 8 weeks after injury, is critical for assessing tissue regeneration, neuronal survival, and the overall recovery of neural function. Paraffin sections of spinal cord samples were co‐stained with GFAP, β3‐tubulin, and Nestin to evaluate the proliferation of abnormal astrocytes. BrdU was used to evaluate the proliferation of neural cells. By intravenously injecting BrdU into mice 1 h prior to specimen collection, this marker of proliferation was able to be introduced into the mice, as demonstrated by the following calculation:

(1) $$\begin{array}{c} \mathrm{Injection}\;\mathrm{Volume}\left(\mathrm{mg}\right)=\mathrm{Body}\;\mathrm{Weight}\left(g\right)\times \mathrm{BrdU}\;\mathrm{Concentration}\left(\mathrm{mg}\;mL^{-1}\right) \end{array}$$

By staining Iba1, a marker for microglia/macrophages, An experiment was conducted to evaluate neuroglial inflammatory stress. In this experiment, antibodies against Iba1 were utilized. Moreover, co‐staining with CD68/iL‐1 was performed to evaluate the inflammatory status post‐injury. The stained specimens were observed under an optical microscope and analyzed using Imaris (Bitplane Co., Ltd., Zurich, Switzerland) and Image J (National Institutes of Health, Bethesda, United States).

### Whole Transcriptome Sequencing

Tissues were processed for immunofluorescence at 8 weeks post‐injury. Whole transcriptome sequencing was extracted from each thymic sample using an RNA Mini Kit (Qiagen, Germany). The raw sequencing data underwent quality control assessment using Skewer and FastQC v0.11.2. The sequencing reads were 2 × 150 bp in length. Transcript abundance was quantified in Fragments Per Kilobase of exon model per Million mapped reads (FPKM) using Perl.

### Preprocessing of Single‐Cell RNA‐Sequencing

The MobiVision software pipeline (version 1.1) was provided by MobiDrop. Subsequently, the unique molecular identifier (UMI) count matrix was processed using the R package Seurat (version 4.0.0).^[^ ^40^, ^41^ ^]^ After QC, 9053 single cells were included in downstream analyses. Library size normalization was performed using the NormalizeData function in Seurat, employing the global‐scaling normalization method “LogNormalize” and log‐transformation. Top variable genes across single cells were identified using the method described by Macosko et al.^[^ ^42^ ^]^ Cell types were inferred using the R package SingleR^[^ ^43^ ^]^ with the reference transcriptomic dataset “immgen.”^[^ ^44^ ^]^

### Statistical Analysis

All experiments were independently repeated at least three times, and all final data were presented as mean ± SD. Statistical analysis and graphing were performed using GraphPad Prism 9.0 (GraphPad Software Inc., CA, USA). Student *t*‐tests were used for comparisons between two groups, while one‐way ANOVA was used for comparisons involving three or more groups. Statistical significance was denoted by *p*‐values of 0.05(*),0.01 (**), and 0.001 (***) were statistically significant. The sample sizes for each analysis were: Functional behavior evaluation: *N* = 6 per group; cell labeling and immunofluorescence evaluation, whole transcriptome sequencing, and single‐cell RNA‐sequencing: *N* = 3 per group.

## Conflict of Interest

The authors declare no conflict of interest.

## Author Contributions

Q.L., S.G., and Y.Q. contributed equally to this work, Q.L., S.G., and Y.Q. performed materials characterization, cell culture, in vivo experiments. N.S., Z.W., and B.W. provided assistance in histology analysis. Q.L., W.Y., J.Y., Q.S., Y.D., and L.C. analyzed the data and wrote the manuscript with help from all authors. B.S., J.W., and W.C. revised the manuscript. W.J., Y.L., and W.C. directed and supervised the study. All authors discussed the results and commented on the manuscript.

## Supporting information

Supporting Information

## Data Availability

The data that support the findings of this study are available from the corresponding author upon reasonable request.
