# Supplementary material for: Regulating Astrocytes via Short Fibers for Spinal Cord Repair
Source: Adv Sci (Weinh). 2024 Aug 9;11(41):2406742. doi: 10.1002/advs.202406742 (PMC11538653; doi:10.1002/advs.202406742)
Supplement: Supplementary file 1 — Supporting Information [file ADVS-11-2406742-s001.docx]

**Regulating Astrocytes *via* Short Fibers for Spinal Cord Repair**

***Qianyi Li, Shuaiyun Gao, Yang Qi, Nuo Shi, Zhenzhen Wang, Qimanguli Saiding, Liang Chen, Yawei Du, Bo Wang,Wenfei Yao, Bruno Sarmento, Jie Yu, Yiming Lu^,*^, Juan Wang^*^, Wenguo Cui^*^***

**Q. Li, W. Cui, Q. Saiding, L. Chen, Y. Du, J. Wang**

Department of Orthopaedics, Shanghai Key Laboratory for Prevention and Treatment of Bone and Joint Diseases, Shanghai Institute of Traumatology and Orthopaedics, Ruijin Hospital, Shanghai Jiao Tong University School of Medicine, Shanghai, P. R. China

E-mail: wgcui80@hotmail.com (W.Cui), juanwang1006@126.com (J.Wang)

**Q. Li, Y. Qi, S. Gao, W. Yao, J. Yu, Y. Lu**

**Department of Emergency, Ruijin Hospital, Shanghai Jiaotong University School of Medicine, Shanghai,** P. R. China.

E-mail: **luyiming@rjh.com.cn** (Y.Lu)

**Q. Li, S. Gao, B. Wang, Y. Lu**

**Pˆole Sino-Franc¸ais de Recherches en Sciences du Vivant et G´enomique, Shanghai,** P. R. China.

**Q. Li, S. Gao, B. Wang, Y. Lu**

International Laboratory in Cancer, Aging and Hematology, Shanghai Jiao Tong University School of Medicine/Ruijin Hospital/CNRS/Inserm/Cote d'Azur University, Shanghai, P. R. China

1. **Shi, Z. Wang**

Peterson's Lab, Shanghai, P. R. China

**Y. Lu**

**Division of Critical Care, Nanxiang Hospital of Jiading District, Shanghai,** P. R. China

1. **Sarmento**

I3-Instituto de Investigação e Inovação Em Saúde and INEB-Instituto de Engenharia Biomédica, Universidade Do Porto, Rua Alfredo Allen 208, 4200-135, Porto, Portugal; IUCS-Instituto Universitário de Ciências da Saúde, CESPU, Rua Central de Gandra 1317, 4585-116, Gandra, Portugal.

**
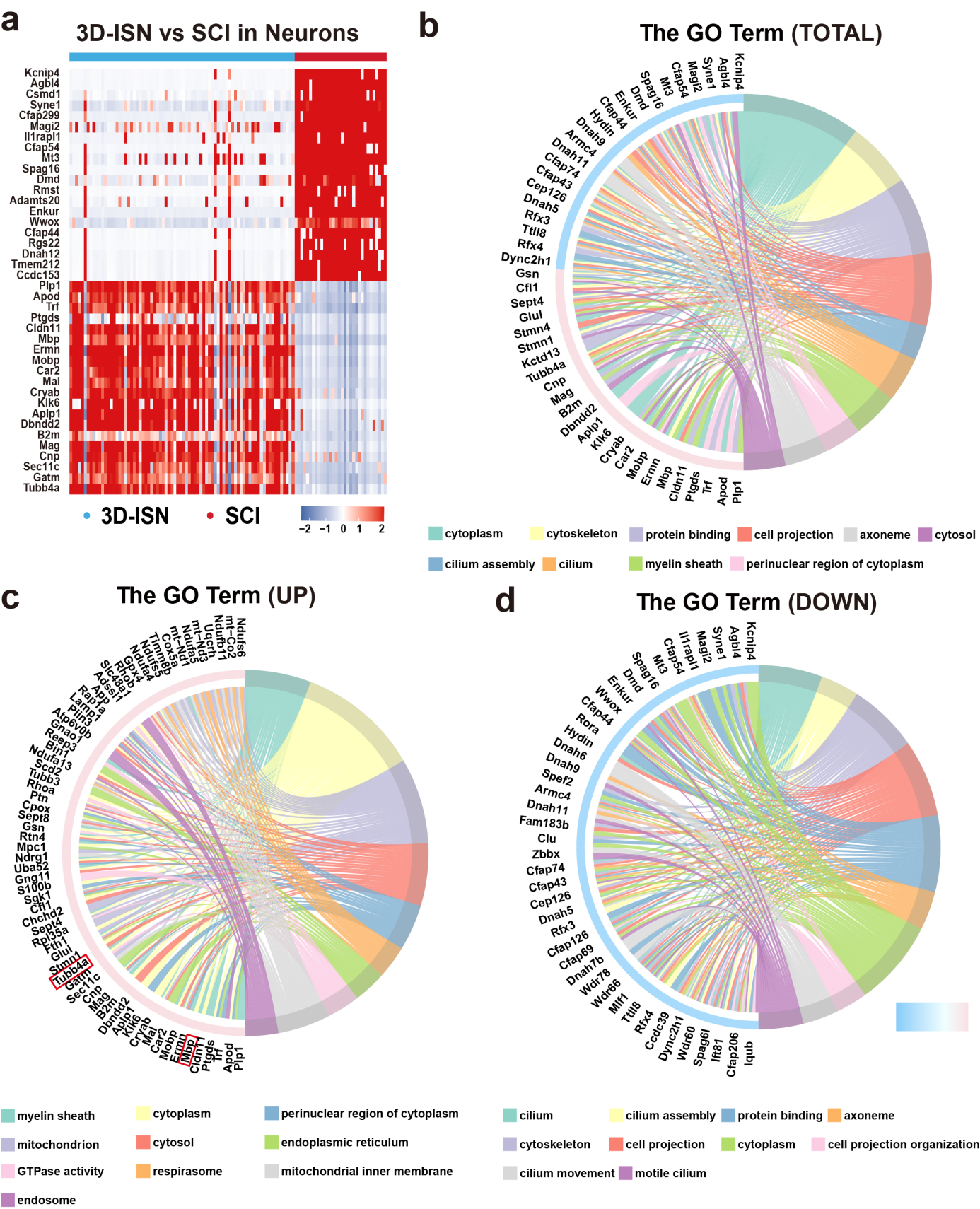
**

**Figure S1. Single-cell sequencing analysis in Neurons :** a) Heat map analysis of DEGs of the 3D-ISN group compared to the SCI group in Neurons from single-cell sequencing . c-d) GO plot of Up/down-regulated genes expressed of the 3D-ISN group compared to the SCI group in Neurons (N=3 in each group).


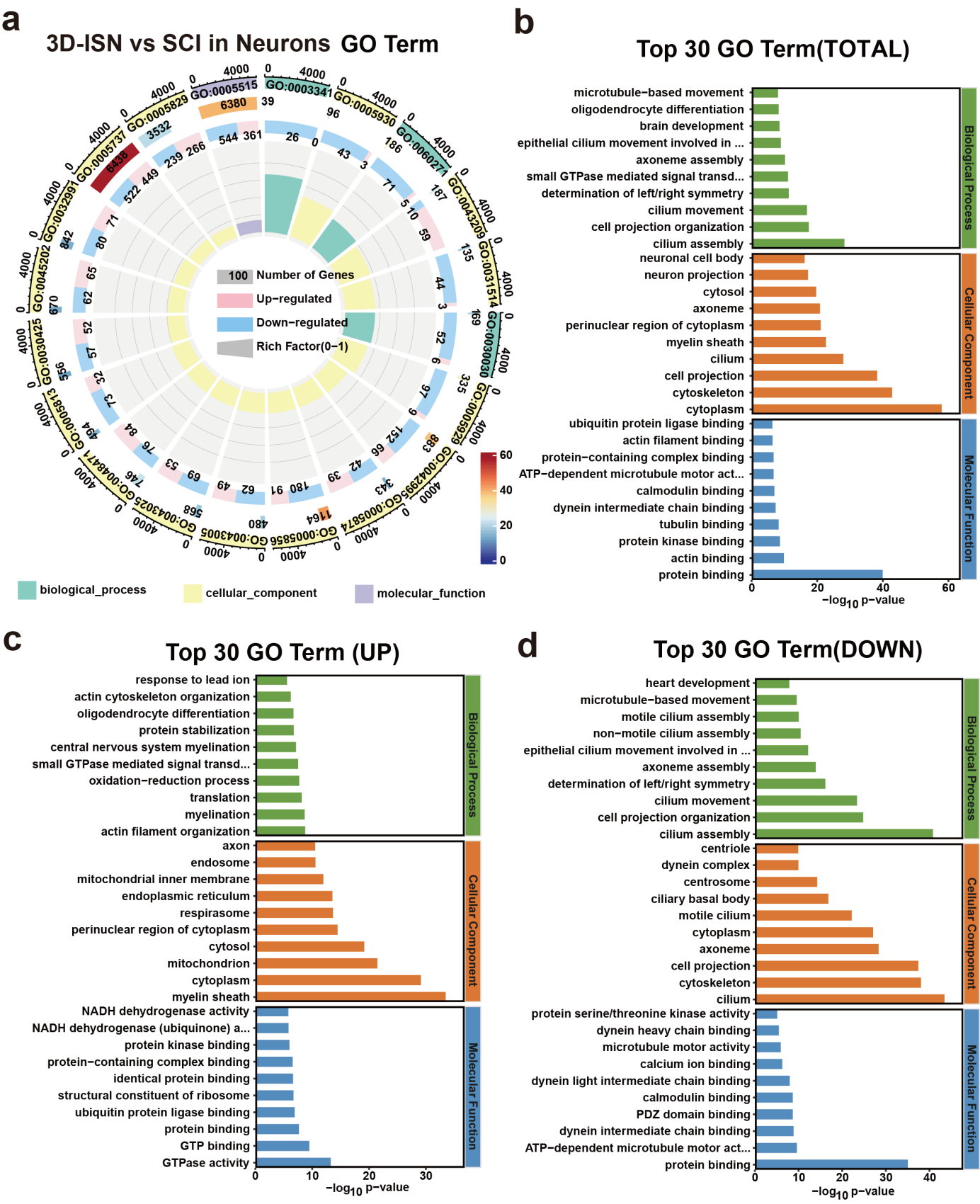


**Figure S2. The GO analysis of the 3D-ISN group in Neurons:** a) GO plot of Up/down-regulated genes expressed of the 3D-ISN group compared to the SCI group in Neurons. b-d) Up/down-regulated GO terms of the 3D-ISN group compared to the SCI group.


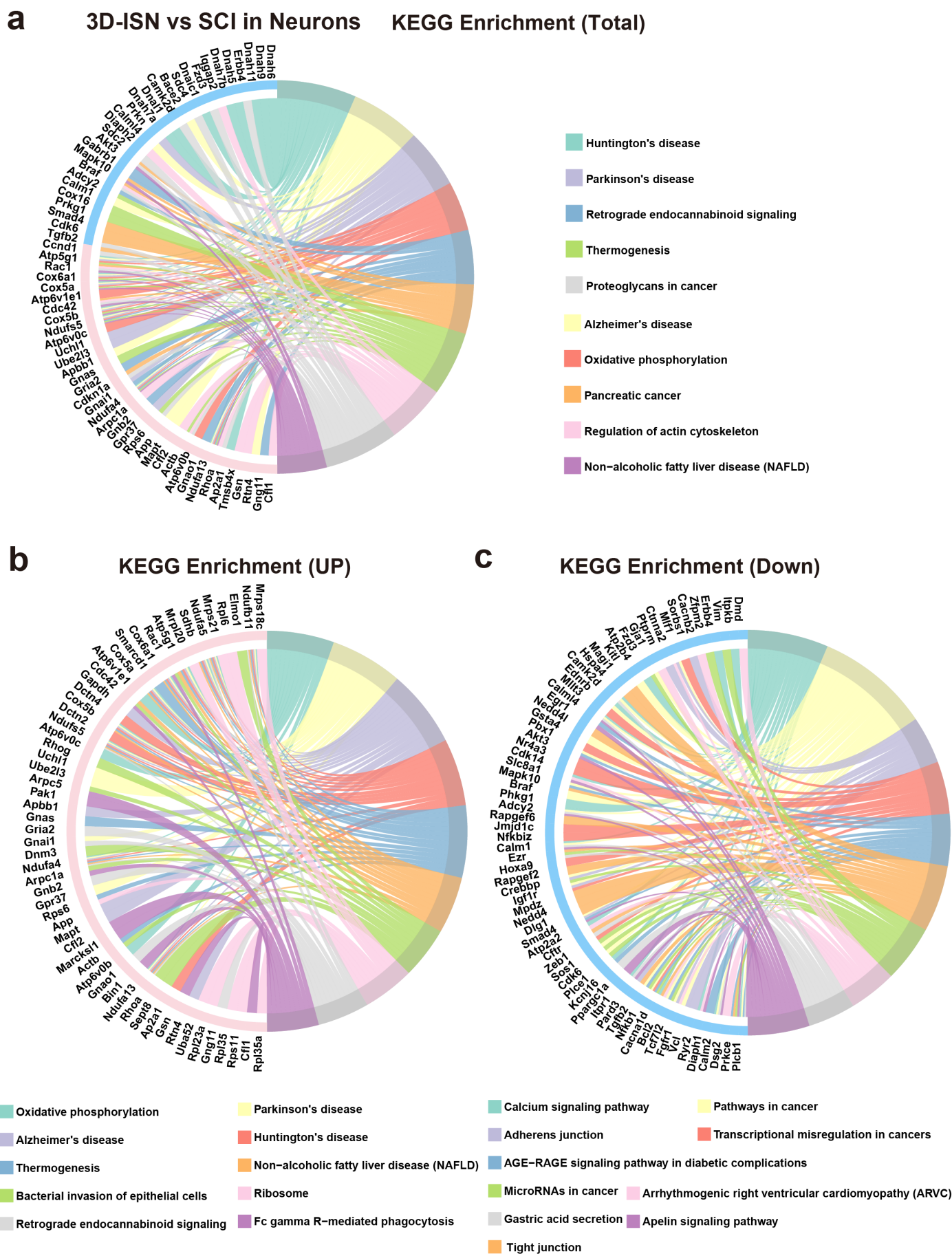


**Figure S3. KEGG analysis between SCI group and Sham group in Neurons.** a-c) KEGG plot of Up/down-regulated genes expressed of the 3D-ISN group compared to the SCI group in Neurons (N=3 in each group).


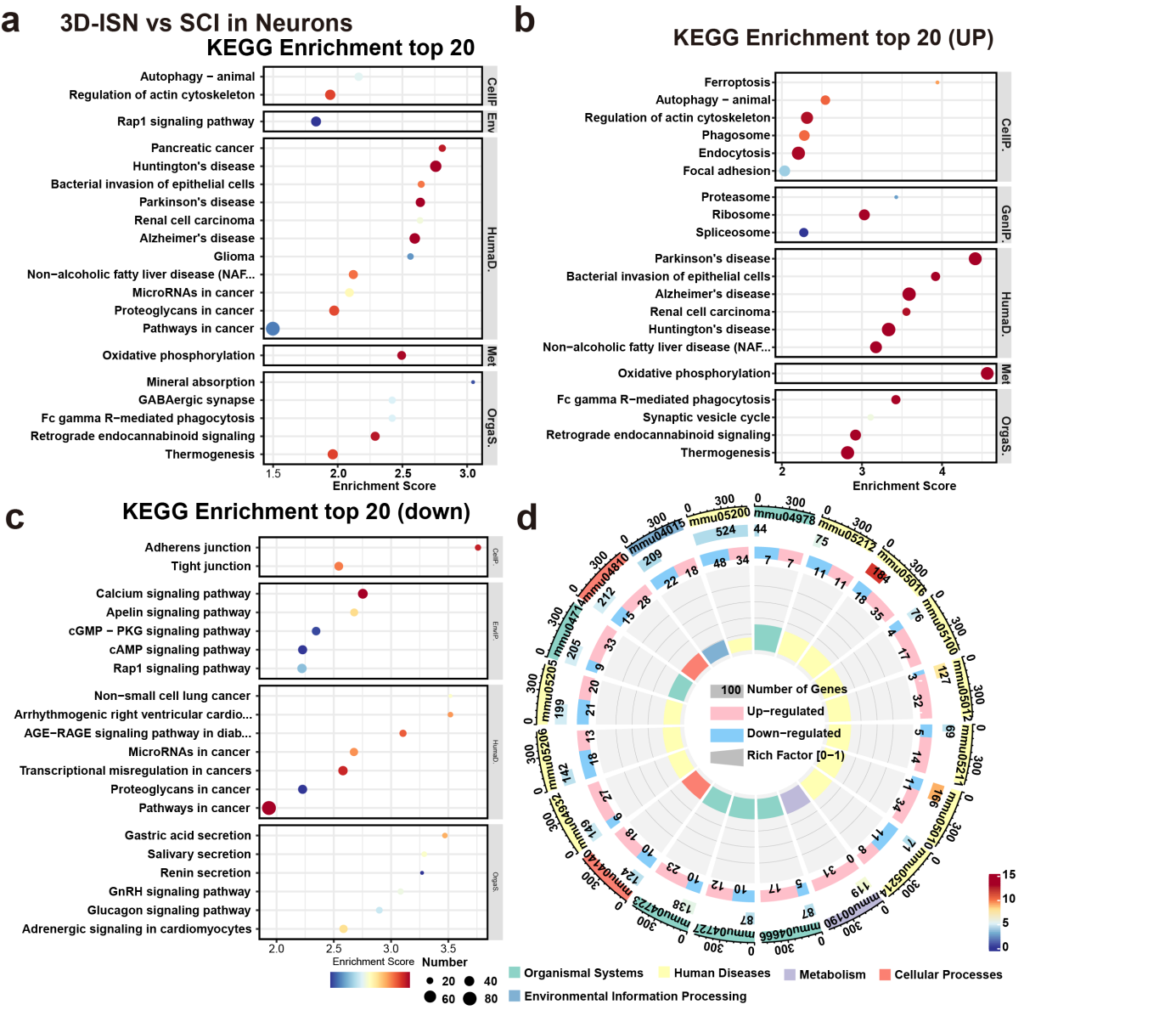


**Figure S4. KEGG enrichment analysis of the 3D-ISN group in neurons:** a-c) Up/down-regulated KEGG enrichment chart of the 3D-ISN group compared to the SCI group. d) The KEGG enrichment between the 3D-ISN group and the SCI group in neurons (N=3 in each group).
